# Comparative Characterization of the Sindbis Virus Proteome from Mammalian and Invertebrate Hosts Identifies nsP2 as a Component of the Virion and Sorting Nexin 5 as a Significant Host Factor for Alphavirus Replication

**DOI:** 10.1128/JVI.00694-18

**Published:** 2018-06-29

**Authors:** Ryan Schuchman, Andy Kilianski, Amanda Piper, Ricardo Vancini, José M. C. Ribeiro, Thomas R. Sprague, Farooq Nasar, Gabrielle Boyd, Raquel Hernandez, Trevor Glaros

**Affiliations:** a U.S. Army Edgewood Chemical Biological Center, Aberdeen Proving Ground, Maryland, USA; b Department of Molecular and Structural Biochemistry, North Carolina State University, Raleigh, North Carolina, USA; c National Institute of Allergy and Infectious Diseases, Laboratory of Malaria and Vector Research, Rockville, Maryland, USA; d U.S. Army Medical Research Institute of Infectious Diseases, Fort Detrick, Maryland, USA; e Excet, Inc., Springfield, Virginia, USA; University of Kentucky College of Medicine

**Keywords:** alphavirus, mass spectrometry, Sindbis virus, viral proteomics

## Abstract

Recent advances in mass spectrometry methods and instrumentation now allow for more accurate identification of proteins in low abundance. This technology was applied to Sindbis virus, the prototypical alphavirus, to investigate the viral proteome. To determine if host proteins are specifically packaged into alphavirus virions, Sindbis virus (SINV) was grown in multiple host cells representing vertebrate and mosquito hosts, and total protein content of purified virions was determined. This analysis identified host factors not previously associated with alphavirus entry, replication, or egress. One host protein, sorting nexin 5 (SNX5), was shown to be critical for the replication of three different alphaviruses, Sindbis, Mayaro, and Chikungunya viruses. The most significant finding was that in addition to the host proteins, SINV nonstructural protein 2 (nsP2) was detected within virions grown in all host cells examined. The protein and RNA-interacting capabilities of nsP2 coupled with its presence in the virion support a role for nsP2 during packaging and/or entry of progeny virus. This function has not been identified for this protein. Taken together, this strategy identified at least one host factor integrally involved in alphavirus replication. Identification of other host proteins provides insight into alphavirus-host interactions during viral replication in both vertebrate and invertebrate hosts. This method of virus proteome analysis may also be useful for the identification of protein candidates for host-based therapeutics.

**IMPORTANCE** Pathogenic alphaviruses, such as Chikungunya and Mayaro viruses, continue to plague public health in developing and developed countries alike. Alphaviruses belong to a group of viruses vectored in nature by hematophagous (blood-feeding) insects and are termed arboviruses (arthropod-borne viruses). This group of viruses contains many human pathogens, such as dengue fever, West Nile, and Yellow fever viruses. With few exceptions, there are no vaccines or prophylactics for these agents, leaving one-third of the world population at risk of infection. Identifying effective antivirals has been a long-term goal for combating these diseases not only because of the lack of vaccines but also because they are effective during an ongoing epidemic. Mass spectrometry-based analysis of the Sindbis virus proteome can be effective in identifying host genes involved in virus replication and novel functions for virus proteins. Identification of these factors is invaluable for the prophylaxis of this group of viruses.

## INTRODUCTION

Epizootics of reemerging and novel pathogenic viruses have increased in recent years, causing infections of susceptible human populations in some of the poorest countries in the world and imposing constant risks on public health. With increasing weather events, travel, and sexual transmission, an even larger population may be at risk. The alphavirus genus (Family Togaviridae) includes emerging and well-characterized vector-borne diseases, including eastern, western, and Venezuelan equine encephalitis viruses (EEEV, WEEV, and VEEV), Chikungunya virus (CHIKV), Ross River virus (RRV), and Mayaro virus (MAYV). Sindbis virus (SINV), the prototype of this group, is nonpathogenic (1). These pathogens are vectored in nature from zoonotic reservoirs, through mosquitoes or ticks to humans, and cycle from vertebrate to invertebrate hosts (arboviruses) (2). Upon emergence, these viruses spill over into the human population and cause severe disease. Viruses are obligate parasites of their hosts and require the host's biosynthetic machinery to propagate all virus infections, necessitating prophylactic treatments that do not harm the host. With few exceptions, all arbovirus diseases lack effective vaccines and antivirals (3). Host-based therapeutics are thought to be alternative avenues, but identifying targets for this approach requires a detailed understanding of how these viruses interact with their host throughout the replication cycle (4, 5). Identification of host cofactors to virus enzymes or specific host factors involved in viral replication and packaging is challenging. The research presented here focused on the identification of host proteins within SINV particles. This was done in an effort to identify host factors associated with virus replication and address any functional relevance these specific proteins might have in viral propagation. In an effort to determine if mass spectrometry could (i) identify host proteins in the virus particle that could participate in subsequent infections or (ii) identify host proteins trapped in the particle during the process of maturation, virus was grown in three different vertebrate and one invertebrate host cell line.

Because of this parasitic characteristic, it was originally thought necessary to specifically target virus factors for antiviral and vaccine development. However, this approach has not always been successful, so methods to target host factors required for viral propagation have been sought. Certain DNA and RNA viruses have long been known to package host factors into virus-encoded progeny to participate in subsequent infections. It is well established that retroviruses package host lysyl-tRNA synthetase (LysRS) by contact with Gag, which targets tRNA to prime transcription to DNA upon infection of a new host (6). Host factors have also been previously observed in virus particles, such as influenza virus (7), vesicular stomatitis virus (8), vaccinia virus (9), and herpes simplex virus (10) (reviewed in reference 11).

Infection by SINV begins with attachment of E2 protein to specific cell receptor(s) (12). The formation of this complex is proposed to induce conformational changes within the virus that produce a pore within the membrane and facilitates the release of the positive-sense, single-stranded viral RNA directly into the cell (13–16). In the cytoplasm, the RNA is then translated into four nonstructural proteins, nsP1, nsP2, nsP3, and nsP4 (17). Together these proteins, in association with unknown host proteins, comprise the replication complex, which is found associated with the plasma membrane in vertebrate cells (18). Transcription of viral RNA produces polycistronic full-length and subgenomic 26S RNAs. In both vertebrate and invertebrate cells the nonstructural proteins are translated from full-length RNA, while the structural proteins are translated from the subgenomic 26S RNA. The 26S RNA encodes the proteins in the order capsid (C), PE2 (processed to E3 and E2), 6K/TF (transframe), and E1, assembled in the endoplasmic reticulum (ER) and processed by host signalase and furin proteins (19). The matured virus from all host species contains the structural proteins C, E1, and E2. 6K is not found associated with Sindbis virions, but liquid chromatography-tandem mass spectrometry (LC-MS/MS) analysis of the 6K chimera frameshift TF protein have identified the latter in Sindbis particles (20). In vertebrate hosts, the glycoproteins mature via the exocytic pathway and are delivered to the plasma membrane. Concurrently, the capsid protomers are preassembled into viral nucleocapsids with full-length RNA found within the cytoplasm and are transported to the plasma membrane, where they acquire their glycoprotein-modified outer shell (12). The virus fully matures as E1- and E2-modified plasma membrane surrounds the nucleocapsid (NC) core and is extruded into the surrounding environment (21, 22) in a process that excludes the preponderance of host plasma membrane proteins (23). In invertebrate cells the virus takes an alternative path to maturation. Translation and replication complexes are housed in cytoplasmic endosomes, termed virus factories, where all assembly mechanics occur. Fully formed virions are then secreted into the media by fusion of the endosomes with the cell plasma membrane (24, 25). In both hosts, the final virion is composed of each of the structural proteins C, E1, and E2 in a 1:1:1 stoichiometric ratio, producing a highly stable and symmetrical virus (26). The mature structure contains an outer protein shell composed of the glycoproteins E1 and E2 separated by a host-derived membrane from the inner NC core. The lipid composition of the membrane will vary between vertebrate or invertebrate hosts, especially with respect to cholesterol, which is not synthesized by the class Insecta (27, 28). In nature, arboviruses are maintained in a complex cycle involving infections between the virus vertebrate reservoir, the insect vector, and the human host and can replicate in many host backgrounds (29). For this reason, we used cell lines of various tissue types and host backgrounds to characterize the complement of host cell-derived proteins. To determine the host protein content of the model alphavirus particle, SINV, a mass spectrometry-based approach was utilized to identify host proteins within virions that might not be resolvable using other methods (30). Additionally, mass spectrometry can allow for the identification of proteins that are specifically associated with the virus structure or alternatively have become trapped or assembled into the structure during the process of maturation.

In this study, highly purified SINV preparations derived from mosquito and mammalian cells were analyzed using LC-MS/MS to determine if previously undetected host proteins were packaged into virus particles. More than 124 unique host proteins were discovered in virus replicated in the human cell backgrounds of HEK293 (human embryonic kidney cells) or HepG2 (human hepatic cancer) cells, and in hamster BHK21 (baby hamster kidney) cells more than 38 host proteins were identified. When the host protein profiles between mammalian species were cross compared, eight proteins were found in common. SINV was also prepared in a mosquito C7-10 Aedes albopictus cell line, an Aedine cell line which exhibits both lytic and persistent infections (31). Although more than 50 host proteins were identified in the mosquito background, phospholipid scramblase was the most abundant and interestingly was the only protein found in both the invertebrate and vertebrate backgrounds. A detailed mapping of the viral proteome revealed a nearly 75% coverage of SINV's structural polyprotein, which contains peptides detected for capsid, E3, E2, E1, and TF but not 6K. An unexpected and intriguing finding was that peptides from SINV's nonstructural polyprotein were also detected but specifically only for nsP2, the viral protease, which was not previously detected (32–34).

In order to determine a related function of these coidentified proteins during SINV replication, we attempted to generate stable homozygous knockouts (KO) of each of the eight identified host proteins in HEK293 cells. We were able to obtain homozygous knockouts for sorting nexin 5 (SNX5) and RNA binding protein 3 (RBM3), while others were lethal. Using these knockout cell lines, we observed a significant suppression in Sindbis virus production compared to that of wild-type HEK293 cells. To confirm this observation, we also performed the same analysis using Mayaro and Chikungunya viruses. Viral replication was also inhibited for both of these viruses. To the best of our knowledge, this is the first report of a sorting nexin, SNX5, playing a critical role in alphavirus replication. Collectively, this LC-MS/MS approach has expanded the tools that can be used for understanding the mechanics of alphavirus replication while also identifying novel host targets with therapeutic potential.

## RESULTS

### Determination of viral purity.

**FIG 1:**
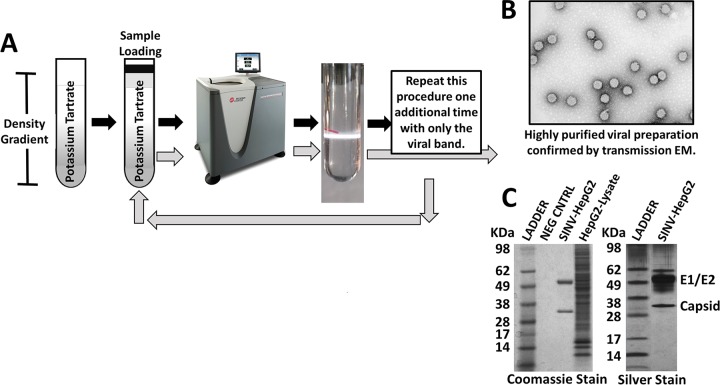
Sindbis virus (SINV) virion purification strategy and production flowchart. (A) Clarified viral preparations were purified over two potassium tartrate density gradients done in series using isopycnic ultracentrifugation and a final wash and pellet step. Negative controls (uninfected cell culture supernatant) were processed in parallel with the virus preparations. (B) Transmission electron micrographs were taken of the purified virions to confirm efficiency of this enrichment procedure. (C) An SDS-PAGE gel was stained with silver or with Coomassie blue for each of the viral preparations. HepG2 preparations are presented here and are representative of all viral preparations from all host cellular backgrounds.

In order to effectively analyze the virus-associated host protein content, ultrapure preparations of virions were prepared. Host background contamination from cellular vesicles or debris not specifically incorporated needs to be eliminated by the virus purification procedure prior to mass spectrometry analysis. The high particle/PFU ratio of SVHR, its structural stability, and known exclusion of host proteins from the virus-modified membrane makes this an ideal experimental model. First, virus was grown and supernatant collected from infected and noninfected cells. The supernatant was harvested from a single-cycle infection prior to cell lysis; crude viral preparations were clarified by low-speed centrifugation to remove large complexes and particulates. Negative-control fractions were run through the same process as virus-containing fractions. The clarified material was then run through two serial potassium tartrate density gradients using isopycnic ultracentrifugation. Virus from the second purification was then pelleted through phosphate-buffered saline (PBS), and the pellet was washed as the final purification step. To determine if this purification scheme (Fig. 1A) resulted in visually pure preparations of virus particles free of contaminating organelles or membrane fragments, fractions were visualized using transmission electron microscopy by negative stain (Fig. 1B). The electron micrographs of the three-step purified SINV preparations visually show high-purity virus preparations, with little evidence of cell debris or other cell-associated membrane-bound vesicles. To determine if there was any other protein contamination present in the preparations, each purified preparation was resolved on an SDS-PAGE gel and stained with silver or Coomassie blue stain as described above. Similar to the transmission electron micrographs, the SDS-PAGE analysis also confirmed that there was no major protein impurities in the preparations (Fig. 1C). The primary bands in the viral preparations were from the virus structural proteins E1, E2, and capsid.

### Host and virus protein identification and functional analysis of purified SINV virions.

Following the confirmation of the purity of the virus preparations, SINV and negative-control preparations were analyzed, in parallel, using a filter-aided sample preparation (FASP) LC-MS/MS method. Viral preparations and negative controls from BHK21 (hamster; Cricetulus griseus), C7-10 (mosquito; Aedes albopictus), HEK293 (human), and HepG2 (human) cell host backgrounds were analyzed to determine if any host proteins could be detected. Each sample was analyzed in technical triplicate on a virgin C_18_ high-performance liquid chromatography (HPLC) column to ensure that carryover from sample to sample would not result in false host protein identifications. LC-MS/MS spectral data were searched using the SEQUEST algorithm in Proteome Discoverer 1.4 (Thermo Fisher) to identify peptides and align them to known host proteins from each background. Each purified virus preparation yielded unique host protein identifications, with only a limited number of host proteins being identified in the negative controls. Keratin was often identified in the negative control and was not considered for functional interpretation because it is shed by cells in culture or introduced during sample processing. In the human backgrounds, 124 host proteins were identified, 91 in HepG2 cells and 58 in HEK293 cells (see Table S1 in the supplemental material). Using the PANTHER classification to characterize these protein identifications, a significant portion of the proteins fell into three major functional classes: binding, catalytic activity, and receptor activity.

Compared to the human proteins found associated with SINV, an additional 38 host proteins were identified from the BHK21 hamster background and 51 proteins from the C7-10 mosquito background (Table S1). It is of note that mammalian and insect cells do not always express mammalian protein orthologs. Both hamster and human genomes and proteomes are well characterized; however, the same level of characterization does not exist in the mosquito host Aedes albopictus, as the genome sequence was very recently published (35). To analyze the raw data obtained from the C7-10 cell background, a curated draft proteome from Aedes albopictus was utilized (see Materials and Methods). Many of the proteins identified in the C7-10 background classify in similar functional categories and classes as the proteins identified in the mammalian backgrounds. Interestingly, for the mosquito background, phospholipid scramblase was overwhelmingly represented compared to the other mosquito host proteins identified. Scramblase was detected as 8 unique peptide sequences a total of 57 times (PSMs) for total sequence coverage of 30% (Table S1).

### Coinciding host protein signatures between human, hamster, and mosquito cell backgrounds.

**FIG 2:**
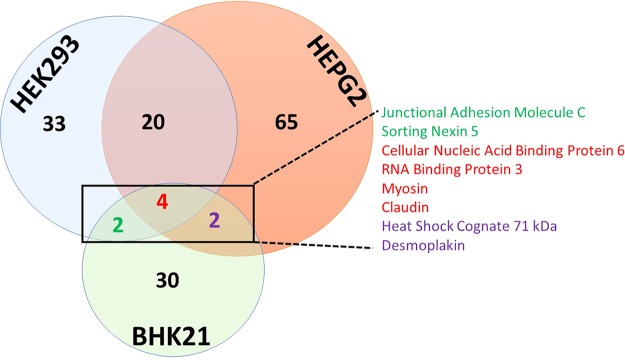
Comparison of the mammalian viral host proteomes.

**FIG 3:**
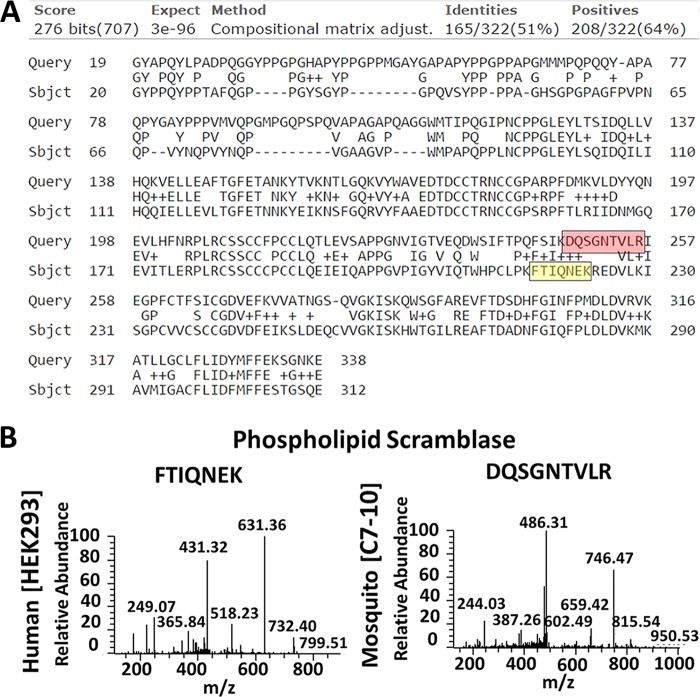
Mosquito phospholipid scramblase detection. (A) The NCBI BLAST alignment tool was used to align the protein sequence of phospholipid scramblase for Aedes albopictus and Homo sapiens. Query indicates the Aedes albopictus sequence (accession number 604777565), and Sbjct indicates the Homo sapiens sequence (accession number 10863877). Peptide sequences presented below the alignment are highlighted in red (mosquito) and yellow (human). (B) MS2 spectra used to identify phospholipid scramblase in HEK293 and C7-10 cells.

When comparing human virus preparations (HEK293 versus HepG2) only to each other, we observed a 34% overlap or identified 20 proteins (Fig. 2). Given that the limiting factor, which dictates detection by LC-MS/MS, is dynamic range and instrument sensitivity, the host proteins that were commonly detected between each preparation were also in the highest abundance (indicated by the total number of PSMs, or ∑#PSMs) within these virus particles. In addition to comparing preparations within the same species, we also compared the host proteins identified across species. Using this strategy, we identified 8 protein signatures that were conserved between hamster and human (Fig. 2). Cellular nucleic acid binding protein 6 (CNBP6), RNA binding protein 3 (RBM3), myosin (MYO), and claudin (CLDN) were present in virus particles grown in all backgrounds. Interestingly, the exact peptide sequence was identified for CNBP6, RBM3, and sorting nexin 5 (SNX5) from all vertebrate-derived samples. When we compared the mammalian backgrounds to the mosquito background, we found that only phospholipid scramblase was conserved. Also of note, we detected that the scramblase peptides overlapped even though only 51% of the amino acid sequence is conserved between mosquito and human proteins (Fig. 3).

### Viral structural and nonstructural proteins identified in purified SINV virions.

In addition to the detailed analysis of the host proteome contained within the SINV virion, the viral proteome within SINV virions was also examined. Raw data gathered from each SINV preparation regardless of host background was pooled to generate a comprehensive sequence map of SINV structural and nonstructural polyprotein coverage (Table S2). For SINV's structural polyprotein, 91 unique peptide sequences were detected a total of 10,833 times, resulting in a total sequence coverage of 74%. Peptides from every processed structural protein were detected except 6K. However, we did detect a single peptide (RLPGEGR), corresponding to TF protein, which is reported to copurify with NC (36). In this study, we also searched our raw data against SINV nonstructural polyprotein. From this analysis, we only detected peptides from nsP2, the virus protease. No peptides from any other nonstructural protein were detected. For nsP2, we detected 20 unique peptide sequences a total of 121 times. Compared to the structural polyprotein, peptides corresponding to nsP2 were detected with a frequency of roughly 1%. To confirm that nsP2 is not copurifying with other structural proteins via protein-protein interactions, we performed an additional high-salt wash in 1 M KCl on viral pellets prepared in the BHK21 background. LC-MS/MS analysis of these preparations still resulted in high sequence coverage of nsP2 and no other nonstructural protein (Table S1).

**FIG 4:**
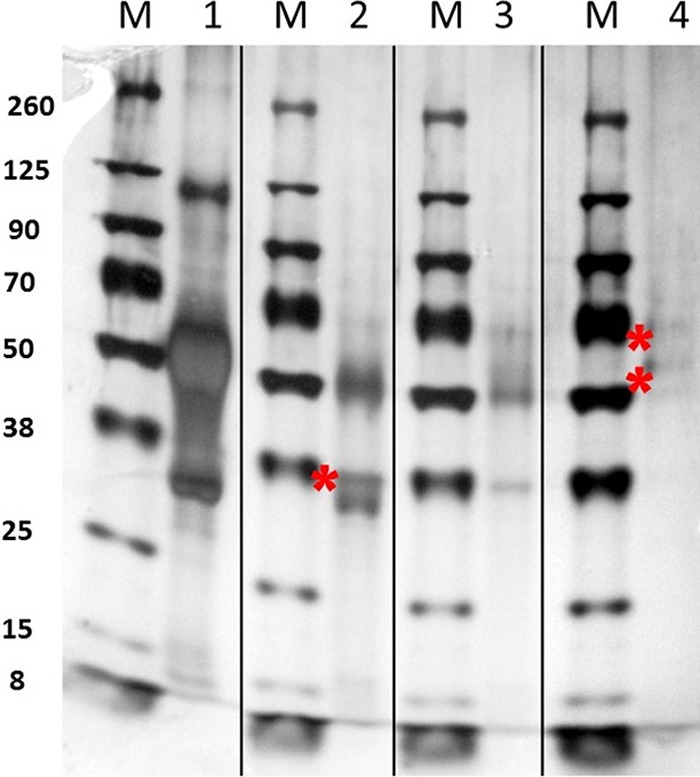
Characterization of SINV nucleocapsid (NC) and glycoprotein fractions. Shown is silver stain of whole virus and NC fractions after increasing treatments of Triton X-100. M, protein marker size (kDa). Lane 1, stained whole SINV; lane 2, whole virus treated with 4% Triton X-100 (NC fraction); lane 3, whole virus treated with 5% Triton X-100 (NC fraction); lane 4, Triton X-100 fractions containing glycoproteins. Note that capsid, E1, and E2 glycoproteins run slower (denoted by an asterisk) after treatment with Triton X-100.

**FIG 5:**
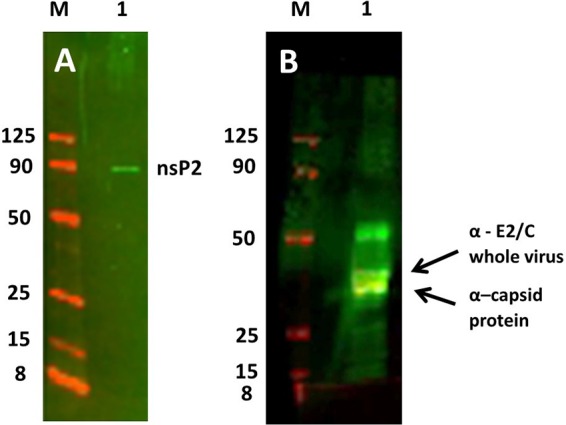
Western blot analysis of NC fractions of purified SVHR. (A) Western blot of whole purified SINV bound to anti-nsP2 Ab using the LI-COR image-capturing system. M is the same marker as that used for Fig. 4; the 260-, 70-, and 38-kDa markers do not fluoresce at the 700- and 800-nm NIR wavelengths used. (B) Western blot of the same fraction as that shown in panel A. Samples were probed first with anti-C Ab (yellow band) and second with anti-whole SINV Ab (green bands). The arrows point to a lower C band, which is the only band that fluoresces with the anti-C Ab, and the upper arrow indicates the upper green C band that only fluoresces with the anti-whole virus Ab. Thus, two capsid bands are resolved in this fraction. E2 is above C in green. Note that E1 does not label with this Ab, while the capsid protein does.

To identify if the nsP2 protein was associated with the SINV outer shell or inner core, we attempted to fractionate the inner core from the outer shell containing the glycoproteins. NC cores were fractionated by treating purified virus particles with 4% or 5% Triton X-100 for 1 h at 37°C to remove the glycoprotein shell, and the fractions were separated by ultracentrifugation. Silver stain of 4 to 12% Bis-Tris gradient gels of the purified fractions (Fig. 4) demonstrate that the NC fractions shown in lanes 2 and 3 are not completely stripped of glycoproteins, showing visible amounts of E1 and E2 on the gel after treatment with 5% Triton X-100. From these silver-stained fractions it could be concluded that the Triton X-100 treatment was not sufficiently stringent to eliminate all of the outer glycoprotein shell from the cores. Additionally, it was clear that treatment of the fractions with Triton X-100 appeared to shift the mobility of C, E1, and E2 proteins, as seen by the appearance of slower-migrating proteins in the 4% Triton X-100 fraction (Fig. 4, lane 2) compared to the 5% Triton X-100 fraction (Fig. 4, lane 3) and the observed E1 and E2 proteins in the glycoprotein fraction seen in Fig. 4, lane 4. Additionally, the doublet C band in Fig. 4 has unlabeled C protein bands similar to those seen in Fig. 5B, lane 1, described below.

Thus, none of the extractions utilizing treatment of the purified virus with 4% Triton X-100 produced fractions pure enough to unambiguously assign the location of nsP2 to a particular subfraction of the virus. However, to definitively identify the virus protein species which were seen in the extracted fractions, Western blotting was done using polyclonal antibodies (Ab) against expressed capsid protein only (36) (raised in rabbits; a gift from S. Mukhopadhyay) or a combination of anti-C Ab and anti-SVHR whole virus (raised in rabbits) and is shown in Fig. 5B. This analysis showed that the anti-capsid Ab only recognized the bottom band of the ∼30-kDa doublet, while both C bands were recognized by the anti-whole SVHR Ab. The anti-SVHR C/E2 antibody recognized only E2 at roughly 50 kDa. These observations indicate that after Triton X-100 treatment, the doublet band running at ∼30 kDa contains 2 forms of C protein. The lower band only binds to the polyclonal anti-C Ab, while the whole SINV Ab virus reacted with both bands of C. The specific lot of anti-whole virus Ab used does not recognize E2, while E1 and C are recognized (data not shown). To positively identify nsP2 protein association with purified SINV, a second blot was made and probed with anti-nsP2 antibody (a gift of R. Hardy), shown in Fig. 5A. Thus, we verified that nsP2 was associated with purified virus but could not definitively identify the location in the particle.

### Virus-associated mammalian host protein requirement for virus replication.

**FIG 6:**
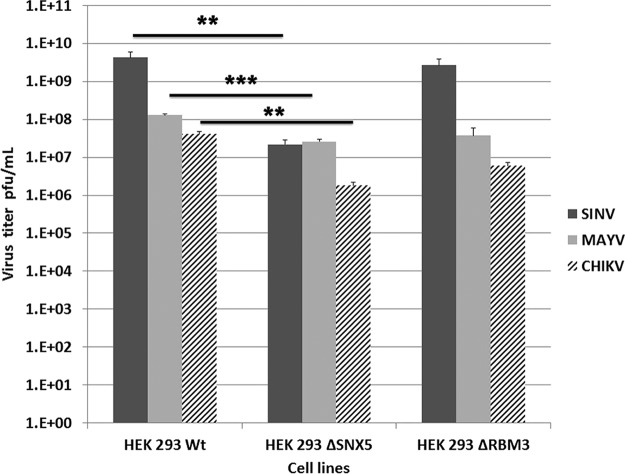
Sindbis virus (SINV), Mayaro virus, and CHIKV production from HEK293 cells. Cells lacking RBM3, SNX5, or wild-type cells were infected with wild-type virus, and infectious virus titers were determined by plaque assay. All three viruses assayed, SINV, MAYV, and CHIKV, replicated to high titers in wild-type HEK293. In contrast, the titers were significantly reduced by >100-fold for SINV (*P* = 0.03), ∼10-fold for MAYV (*P* = 0.003), and 40-fold for CHIKV (*P* = 0.01) in HEK293 ΔSNX5 cells, with *P* values determined by Student's *t* test. For MAYV, reduced viral replication was also observed in HEK293 ΔRBM3; however, the effect was modest, with an ∼5-fold reduction for both SINV and MAYV. CHIKV was more reduced in virus production than MAYV from HEK293 ΔSNX5 and HEK293 ΔRBM3, showing a relative inhibition of 40- and 36-fold, respectively.

Stable knockouts of each of the conserved host proteins were attempted in human HEK293 cells. We were able to obtain homozygous knockouts for sorting nexin 5 (SNX5) and RNA binding protein 3 (RBM3). To establish that this observation is not specific just to Sindbis virus but rather to alphaviruses, we repeated these studies using the New World Mayaro virus, a pathogenic virus (27), and CHIKV, which produces acute febrile polyarthralgia (37). Using these knockout cell lines, we observed a significant suppression in Sindbis, Mayaro, and Chikungunya virus production by standard plaque assay when the cells were grown in the SNX5 or RBM3 knockout cell lines compared to growth in wild-type HEK293 (Fig. 6). Because the virus produced from these knockout cell lines was not as infectious as that of virus produced from wild-type HEK293 cells, as determined by plaque assay on BHK cells, we conclude that these proteins are not absolutely required for infectivity. While SNX5 is important in the efficient maturation of infectious alphavirus, intracellular trafficking of virus was not affected because there was not a significant amount of intracellular virus found in the SNX5-negative cells (data not shown). All three viruses assayed, SINV, MAYV, and CHIKV, replicated to high titers in wild-type HEK293, with titers of 5 × 10^9^, 1 × 10^8^, and 4.2 × 10^7^ PFU/ml, respectively. In contrast, the titers were significantly reduced >100-fold for SINV (*P* value of 0.03), ∼10-fold for MAYV (*P* value of 0.003), and 40-fold for CHIKV (*P* value of 0.01) in HEK293 ΔSNX5 cells (*P* value determined by Student's *t* test). For MAYV, reduced viral replication was also observed in HEK293 ΔRBM3; however, the effect was modest, with an ∼5-fold reduction for both SINV and MAYV. CHIKV was more reduced in virus production than MAYV from HEK293 ΔSNX5 and HEK293 ΔRBM3, showing a relative inhibition of 40- and 36-fold, respectively. Of the two proteins identified, the data demonstrate that the deletion of SNX5 is much more deleterious to alphavirus replication than that of RBM3, thus RBM functions are not further discussed.

## DISCUSSION

The lack of a significant advancement in the development of effective antivirals, vaccines, and investigative tools for the study of pathogenic viruses remains a major gap in the ability to protect susceptible populations from diseases caused by emerging and reemerging pathogens. Basic understanding of virus-host biology remains central to the development of new approaches; therefore, novel techniques must be harnessed to increase this basic scientific knowledge. Here, LC-MS/MS was utilized to determine if the prototypical alphavirus, Sindbis virus, specifically packaged host proteins into its virus particles. A two-stage purification process for SINV virions, followed by a FASP digestion procedure and mass spectrometry analysis, revealed that a variety of host proteins were incorporated into SINV particles from each host background. Central to this approach was ensuring that the viral preparations were free of virion-associated impurities and cellular debris. Transmission electron microscopy and a silver-stained SDS-PAGE gel revealed that our approach yielded high-purity viral preparations that were not contaminated with other cellular debris and membrane fragments and did not have any significant protein contamination (Fig. 1B and C). The observation that 6K was not found associated with the SINV particles is expected, since 6K is not virion associated in SVHR (38). The Aedes albopictus mosquito proteome is not well annotated, so processing of mass spectrometry data from the C7-10 background required our group to generate a manually curated mosquito proteome. Despite this limitation, the functional role of these proteins being associated with SINV replication will aid in experimental validation of their proteomic annotation. In the mammalian backgrounds, the identification of a core group of coinciding host proteins creates strong leads for further antiviral potential (Fig. 3). These proteins should serve as the first set of host proteins interrogated for their functional relevance during the SINV replication cycle and will be discussed first.

In this study, sorting nexin 5 (SNX5) was found to be associated with virus particles when SINV was grown in two mammalian cell lines, HEK293 and BHK21. This host protein has cellular functions which are germane to the replication pathway of SINV, because virus replication and maturation is integrally associated with the endo- and exocytic host pathways. The sorting nexins (SNX) are a family of cytoplasmic proteins associated with a larger protein complex known as the retromer complex (39). This complex plays a vital role in endosomal protein sorting, and this machinery is conserved across all eukaryotes (40). This complex is involved in endosome-to-Golgi trafficking and also in endosome-to-plasma membrane sorting and signaling. These membrane movement events are required to recycle receptors and other plasma membrane components during normal cellular processes. Members of this family contain a phox (PX) domain (41), which is a phosphoinositide binding domain (PIP) (42). Specific SNX5 functions include endosomal sorting via the phosphoinositide signaling pathway and micropinocytosis (43). However, while SNX5 was found only in two of the three mammalian cell lines tested, SNX5 is known to be expressed in HepG2 cells, the third mammalian line (44). Although no SNX5 was found associated with virions produced from HepG2 cells, this may be a result of the level of expression of SNX5 by the HepG2 cells as well as the dynamic range of the LC-MS/MS analysis. This may also explain why SNX5 was not found associated with virions from C7-10 cells, because SNX orthologs are also expressed in the class Insecta (45). An alternative explanation for the lack of detection of SNX5 from insect cells is that alphaviruses mature via secretion from internal vesicles and not through the budding at the plasma membrane (24).

To investigate the requirement of this protein by SINV during replication, stable HEK293 SNX5 knockout cell lines were constructed. By replicating SINV in these cell lines, we were able to show that SNX5 is essential for the growth of Sindbis, Chikungunya, and Mayaro viruses. In the absence of SNX5, replication of virus was found to be significantly inhibited by infectious virus titration by plaque assay of progeny virus. SNX5 has been shown to be critical in the replication of other intracellular pathogens as well, which is relevant to this analysis. Currently, a scenario is beginning to emerge in which the retromer complex is involved in the restriction of pathogens to the host cell by its functional exploitation or repression (46). The best-documented pathogen is chlamydia, a common human bacterial disease. Recently, a pair of studies have elucidated a role for SNX5 as a restriction factor for pathogenesis. The chlamydia intracellular bacteria require membrane structures to replicate and produce infectious progeny within host cells. Both studies determined that SNX5 can negatively impact the membrane rearrangements required by chlamydia to replicate efficiently (47, 48). To counter this restrictive effect, chlamydia sequesters SNX5 in replicative bodies and prevents its action on membrane rearrangements, allowing replication. It is known that membrane spherules are involved in plus-strand RNA replication of alphaviruses (49, 50). It has also been shown that membrane rearrangements and composition are required for assembly of the alphaviruses during virus maturation or budding (23). Additionally, SNX5 was recently been found to colocalize with ebolavirus during the entry phase in an internalization process via micropinocytosis (51). Collectively these observations raise the question of whether membrane reorganization occurs during alphavirus penetration of the host membrane or budding at the plasma membrane by an SNX-mediated process. Although we have shown that SNX5 is essential for alphavirus replication, the exact effect on replication or the mechanism of inhibition is unknown. Other viruses, such as human papillomavirus (HPV) and human immunodeficiency virus (HIV), are known to commandeer the retromer complex for efficient infection via specific mechanisms involving virus entry into the cell (6, 11). Evidence also implicates the retromer complex in a role during hepatitis C virus (HCV) infection as a method to recruit host proteins to the replication complex on a phophoinositol-4-phosphate-rich membrane (52, 53). It is possible that SNX5 can be hijacked by SINV proteins directly or indirectly during particle penetration, early steps of the replication cycle, or, conversely, during particle assembly to induce events necessary for infectious particles to be formed efficiently. Research to uncover the exact role of SNX5 during SINV replication is warranted and could further uncover potential host candidates for antiviral therapeutics.

While SNX5 has been implicated in the replication of infectious alphaviruses, the other proteins identified that were found in common (Fig. 2) in the viral proteome by the reported method have less clear potential roles in SINV replication. We did not detect phospholipid scramblase in two (both mammalian) of the four cell lines investigated, and it was the only host protein which vertebrate- and invertebrate-derived SINV particles displayed in common.

For the coidentified proteins to provide meaningful information on the SINV replication pathway, a putative functional role for the virion-associated proteins must first be identified. Some hypothetical functions of these proteins during SINV replication are suggested below and are amenable to testing. Phospholipid scramblase functions to passively reorganize phosphatidyl inositols (PIPs) interchangeably from the outer and inner leaflet of the cell membranes. During lytic death induced by infection of vertebrates by alphaviruses, membranes are key targets for destruction upon the initiation of cell death by apoptosis and necrosis (54, 55). It has become increasingly documented that PIPs are involved in these processes (56). Thus, PIPs may accumulate during the latter part of the infection as cell lysis approaches. The mosquito C7-10 cell line is the invertebrate cell line which expresses the most SINV-induced lysis of the Aedes-derived lineages (57). Because this process is passive, there is no clear signaling network between scramblase and SNX5. Additionally, phospholipid scramblase 1 has been found to interact with influenza A, suppressing nuclear import and thereby inhibiting virus replication (58).

It is of special interest that junctional adhesion molecule C (JAMC), claudin (CLDN), and myosin are all components of the epithelial cell tight junctions (TJ) that provide a physical barrier between cells (59). These cell structures have been shown to be important for hepatitis C viral entry, replication, dissemination, and exit from the cell (52, 59). Desmoplakin is a component of the desmosome, a cell structure that is also involved in cell-to-cell adhesion. How this protein relates to SINV replication is also not clear. Cellular nucleic acid binding protein 6 (CNBP6) and RNA binding protein 3 (RBM3) both have the ability to bind single-stranded RNA, the species of RNA encoding the SINV genome (60). In fact, RBM3 has already been shown to be associated with the SINV particle (61). Possible functions for these host proteins are to participate in virus translation and/or participate as components of the RNA replication complex (62, 63). These RNA-binding proteins could protect viral RNA from host cell antiviral sensors while also promoting translation of viral polyproteins upon progeny virus entry into the host cell cytoplasm (63, 64). The heat shock proteins HSP70/71 are important throughout viral replication cycles, although it remains unclear if these molecules play a role by being incorporated into alphavirus and SINV virions (65, 66). HSP70 is known to be involved in the assembly of other RNA-containing viruses, such as cucumber necrosis virus (67) and hepatitis C virus (53), and are physically associated with lentivirus particles (68). Another protein chaperone, BIP (HSPA5), belonging to the HSP70 superfamily, has been shown to be required for the assembly of SINV (69, 70). The identification of multiple host factors within SINV particles using this method opens avenues for continued exploration into how these sequestered proteins influence the production of infectious virions or some part of the replication cycle. To definitively identify a role of these proteins, further analysis is required.

Of major significance is the identification of a SINV nonstructural protein, nsP2, associated with the virus particles, an unpredicted and fascinating finding. Alphavirus nonstructural proteins are important for virus replication but have not been found encapsidated into the virus particle. In this study, LC-MS/MS data led to the discovery that nsP2 is incorporated into SINV particles when grown in each of the different host cells tested. It was considered that nsP2 could be copurifying with SINV as a nonspecific contaminant, but its association with the virion was confirmed by performing additional high-salt washes of the virus preparations to eliminate nonspecific binding. To determine whether the nsP2 protein was associated with the outer glycoprotein shell or was held in the nucleocapsid core, the inner and outer shells of purified virus were prepared as described above and dissociated from one another with Triton X-100, and the fractions were run on a 4 to 12% Bis-Tris SDS-PAGE gradient gel. To definitively identify the proteins, Western blot analyses were performed on the nucleocapsid-enriched fraction using anti-whole SINV Ab and anti-capsid Ab. Capsid proteins and E2 were identified in the NC fraction. nsP2 was found incorporated into whole purified virus, although a specific association with the outer shell or inner core could not be established.

This finding is supported by evidence that nsP2 plays a direct or indirect role in RNA encapsidation in Venezuelan equine encephalitis virus (VEEV), another alphavirus (71). This discovery is significant because nsP2 is a multifunctional enzyme previously thought only to function in RNA replication as the viral protease and triphosphatase (NTPase) (72). As the viral protease, its functions range from *cis*- and *trans*-cleavage of viral nonstructural polyproteins to innate immune evasion early in viral replication (73–78). While not all proposed enzymatic roles for nsP2 have been verified or all the identified roles fully understood, one role of this protein is as a transcription factor, i.e., a structural role. The presence of nsP2 in the SINV suggests that this protein has an additional structural role within the SINV virion or is required upon virus entry because it was identified in virions produced in all host backgrounds. The incorporation of an enzyme in a structural role is not novel among viruses. Thymidylate synthase and dihydrofolate reductase are DNA synthesis and replication enzymes encoded by the phage T4. However, they are also essential structural components of the phage baseplate and thus are essential elements for phage infectivity (79). Conversely, by incorporating nsP2 into progeny virions, SINV may use its enzymatic and immune antagonist roles to facilitate the infection at the earliest stages of viral entry, before translation of the plus-stranded viral RNA occurs. It may be involved in recruitment of capsid protein to the RNA nucleation site, thereby participating in NC assembly. Alternatively, nsP2 associated with the genomic RNA could establish the translation complex, which initiates production of the nonstructural proteins and then switches to replicate viral RNA. Any or all of these functions are possible and will be investigated. It is feasible that prophylactic targeting of viral nsP2, which has no other known substrates (17), would serve to disrupt the infection. Functional and structural roles for nsP2 both in *cis* and *trans* imply that the protein can assume multiple conformations and is not amenable to rational design of functional inhibitors for nonenzymatic structures. However, pharmacological targeting of nsP2 is an excellent candidate for an antiviral therapeutic and would fulfill the first tenet of a good therapeutic, virus specificity.

Overall, this study has produced significant results that contribute to the understanding of alphavirus-host biology while identifying SNX5 as a potential target for antiviral therapy. LC-MS/MS analysis of highly pure viral particles determined that SINV specifically incorporates host proteins into progeny virus particles. These proteins have a variety of functions that can be investigated for a role in SINV replication, with a core group of proteins conserved in multiple host backgrounds serving as a logical starting point. Finally, the novel discovery of SINV nsP2 incorporation into virus particles suggests a specific interaction and a possible role for nsP2 during replication or assembly. By interrogating SINV particles for members of the host background proteome, a better understanding of the virus-host interactions necessary for alphavirus entry, replication, protein processing, and particle assembly prior to budding can be established.

## MATERIALS AND METHODS

### Cells, virus growth, preparation, and purification.

The heat-resistant SINV (SVHR) strain used in this study was provided to Dennis Brown (NC State University). This strain was isolated by Burge and Pfefferkorn in 1966 by collecting virus that was resistant to heating to 54°C (80). The choice of strain is important, because this strain produces high titers (10^10^ PFU/ml) of highly infectious and physically stable virus. Chikungunya virus West African strain 37997 was grown and harvested as described in reference 81. Mayaro virus strain CH was utilized for the study and was kindly provided by Scott Weaver at UTMB. Propagation of HepG2 cells (human) and C7-10 cells (mosquito) was as described previously (82). Influenza A/PR/8/34 virus and the MDCK cells used to propagate and titer the virus were a kind gift of Scott Laster, and they were propagated as detailed in reference 83. All cells were obtained from the American Type Culture Collection (Vero, CCL-81; HEK293, CRL-1573; HepG2, HB-8065; BHK21, C-13) except for the C7-10 (Aedes albopictus) cells, which were obtained from internal collections (84). Culture medium from each uninfected cell culture was harvested and served as a negative control. Virus and negative controls were harvested from 10 T-75 flasks (Corning), which produces enough virus to form a visible band in a 30-ml potassium tartrate gradient. Cells were infected at a multiplicity of infection (MOI) of 10 PFU/ml, allowed to replicate for a single cycle, and harvested at 18 h postinfection to ensure that no cell lysis took place. The supernatants were clarified by low-speed centrifugation. Twenty ml of the resulting virus was loaded onto a 15 to 35% linear potassium tartrate gradient and twice purified by isopycnic ultracentrifugation (18 h at 24,000 rpm; Beckman SW-28 rotor). The resulting band of purified virus was collected and washed twice by pelleting the virus in 1× PBS. A sample of the purified population was then visualized by transmission electron microscopy to check for contaminating cellular organelles or membrane fragments as previously described (85). Additionally, to check for copurified protein contaminants, each preparation was resolved on a 4 to 12% Bis-Tris SDS-PAGE gradient gel (Invitrogen) as described previously (86) and stained with silver in the method of Wray et al. (87) or with Coomassie blue. Visualized bands (silver stain only) were excised, and an in-gel digestion was performed as described previously (88) prior to LC-MS/MS analysis for protein identification.

### Sindbis virus particle analysis for nsP2.

Gradient-purified SINV was washed with 1 M KCl to remove nonspecifically bound proteins. Purified SINV particles were then separated into glycoprotein and nucleocapsid fractions by treatment with 4% or 5% Triton X-100 for 1 h at 37°C and pelleted for 45 min through PBS at 45,000 rpm in a Beckman XL 90 centrifuge (89). The supernatant (glycoprotein fraction) was saved and the resulting pellet (nucleocapsid fraction) was washed twice by pelleting through PBS in the same manner. The nucleocapsid fraction was then subjected to LC-MS/MS analysis, SDS-PAGE, or fluorography. Location of the nsP2 protein within the outer or inner shells of the particle was done using staining of 4 to 12% Bis-Tris gradient gels and Western blotting of the glycoprotein and NC fractions of the particle. Virus particles (∼5 μg per lane) were prepared in SDS-PAGE sample buffer supplemented with 5% beta-mercaptoethanol and boiled for approximately 10 min. The samples were run on an SDS-PAGE Bis-Tris 12% gradient gel and transferred to a polyvinylidene difluoride (PVDF) membrane. The membrane was blocked with 5% Carnation nonfat dry milk in PBS for 1 h at ambient temperature. The membrane was then probed for 1 h with a rabbit antibody raised against nsP2 (a gift from R. Hardy) or anti-C protein (a gift from S. Mukhopadhyay) diluted in PBS supplemented with 5% dry milk and 0.1% Tween 20 at ambient temperature. The blot was washed 3 times for 5 min each with PBS supplemented with 0.1% Tween 20 at ambient temperature. Finally, the membrane was probed with an anti-rabbit secondary antibody that was conjugated with IRDye 800CW near-infrared dye diluted in PBS supplemented with 5% dry milk, 0.1% Tween 20, and 0.02% SDS at ambient temperature. The blot was subsequently washed 3 times for 5 min each with PBS supplemented with 0.1% Tween 20 at ambient temperature, washed once with water, dried, and imaged according to LI-COR Odyssey CLx instructions.

### Protein extraction and digestion.

Viral preparations and their respective negative controls were processed for LC-MS/MS analysis using the filter-aided sample preparation (FASP) method as previously described (90). Briefly, following purification the total protein concentration was determined and all preparations were normalized using sterile PBS to 0.5 μg/μl, aliquoted in 10-μg fractions, and stored at −80°C. A total of 10 μg of total protein (20 μl) was mixed 1:1 with MPER (Thermo Fisher) supplemented with 50 mM dithiothreitol (DTT) and heated to 95°C for 10 min. Once cooled to room temperature, the samples were mixed with 200 μl of UA (8 M urea, 100 mM Tris-HCl, pH 8.5), placed over a 30-kDa filter spin column (Ultracell YM-30; Millipore), and centrifuged at 14,000 × *g* for 30 min at room temperature to collect all proteins on the filter membrane. Denatured and reduced proteins were then alkylated by adding 100 μl of IAA solution (0.05 M iodoacetamide in UA) to each filter and incubating at room temperature in the dark for 20 min. Following alkylation, the samples were centrifuged at 14,000 × *g* for 20 min to remove the alkylation solution. Each sample next was washed three times with 100 μl of UA and then three times with 100 μl of 100 mM triethylammonium bicarbonate (TEAB). Centrifugation at 14,000 × *g* for 30 min was used to remove each wash, including the final wash. To digest the captured protein, 100 μl of a trypsin digestion solution (10 μg/ml in 100 mM TEAB) was placed on each membrane and incubated in a sealed tube overnight at 37°C with shaking. After incubation, the peptides were collected for LC-MS/MS analysis in a clean tube by centrifuging each tube for 30 min at 14,000 × *g*. The membrane was washed by centrifugation one time with 50 μl 100 mM TEAB and once with 50 μl of 0.5 M NaCl. All washes were collected and pooled with the final peptide eluate. Each sample was then acidified using 10% trifluoroacetic acid until the final pH was roughly 2 to 3. Prior to mass spectrometric analysis, each sample was desalted using C_18_ desalting columns (Thermo Fisher) according to the manufacturer's directions.

### LC-MS/MS analysis.

Tryptic peptides were analyzed (technical triplicates) on an Orbitrap ELITE mass spectrometer coupled with the Easy-nLC II liquid chromatography pump system. Dried peptides were reconstituted in 3% acetonitrile–0.1% formic acid and resolved on a virgin Picofrit HPLC column (15 cm; 75-μm inner diameter; packed with 5 μm BioBasic C_18_ particles at 300 Å; New Objective) using a 130-min multistep gradient (0 to 5 min, 5 to 10% buffer B; 6 to 110 min, 10 to 35% buffer B; and 111 to 130 min, 35 to 95% buffer B). For the gradient, the A buffer is 3% acetonitrile–0.1% formic acid and the B buffer is 95% acetonitrile–0.1% formic acid. Orbitrap MS1 scans were performed at a resolution of 120,000 at 400 *m/z*, with a scan range of 110 to 2,000 *m/z*. The top 20 precursors were selected for MS2 data-dependent fragmentation. An MS2 spectrum was acquired using ion trap scanning in normal mode (top 20 method). The minimum signal required to trigger a data-dependent scan was 5,000. Collision-induced dissociation (CID) was used to generate MS2 spectra with the following settings: normalized collision energy, 35%; default charge state, 2; isolation width, 2 *m/z*; activation time, 10 ms. The AGC target was set to 1 × 10^6^ for MS and 5 ×10^4^ for MS/MS, with a maximum accumulation time of 200 ms. Dynamic exclusion was set for 60 s for up to 500 targets with a 5-ppm mass window. A lock mass of 445.120025 was used for internal calibration to improve mass accuracy.

### Mass spectrometry data processing.

Spectral data were processed using Proteome Discoverer 1.4 with the SEQUEST search algorithm against a Sindbis virus polyprotein database (Uniprot ID P03317) merged with either Homo sapiens (RefSeq TaxID 9606) or Cricetulus griseus (RefSeq TaxID 10029). The Aedes albopictus-deduced proteome (35) data were downloaded from VectorBase (91). Additional peptides were downloaded from the NCBI TSA database. The nonredundant proteomes were organized on Excel spreadsheets (92) and annotated as described previously (93). The resulting FASTA file was merged with the SINV polyprotein and used to search against the SINV mosquito preparations. Dynamic modifications were set for carbamidomethylation of cysteine [+57.02 Da], oxidation of methionine [+15.99 Da], and N-terminal acetylation [+42.011 Da]. MS/MS spectra were searched with a precursor mass tolerance of 10 ppm and a fragment mass tolerance of 0.6 Da. Trypsin was specified as the protease, with the maximum number of missed cleavages set to 2. A false discovery rate was calculated using PERCOLATOR and was set at <1% to score high-confidence peptide identifications. Grouping and functional analysis were performed using the PANTHER classification system for the human background, using only each protein's accession number (94).

### Phenotypic studies with stable KO HEK293 cells.

HEK293 knockout (KO) cell lines were purchased from Applies Biological Materials, Inc. (Richmond, BC, Canada). SNX5 and RBM3 KO was confirmed by Sanger sequencing. Cell lines were propagated at 37°C with 5% CO2 in Dulbecco's minimal essential medium (DMEM) containing 10% (vol/vol) fetal bovine serum (FBS), sodium pyruvate (1 mM), 1% (vol/vol) nonessential amino acids, and 50 μg/ml gentamicin. Briefly, HEK293 monolayers were infected at equal MOIs (approximately 10 PFU/cell) of SVHR, CHIKV, or MAYV and allowed to incubate for 20 h. Supernatants were titrated on BHK cells by the standard plaque assay to determine the virus titer. As a measure of the knockout cells' ability to replicate an unrelated enveloped virus, influenza A/PRF/8/34 virus was used to infect the wild-type and ΔSNX5 HEK293 cells. Infectious virus titer was determined by plaque assay. Both ΔSNX5 HEK293 cells and wild-type cells produced an equivalent amount of infectious virus after 24 h.

## Supplementary Material

Supplemental material
