# Supplementary material for: Comparative Characterization of the Sindbis Virus Proteome from Mammalian and Invertebrate Hosts Identifies nsP2 as a Component of the Virion and Sorting Nexin 5 as a Significant Host Factor for Alphavirus Replication
Source: J Virol. 2018 Jun 29;92(14):e00694-18. doi: 10.1128/JVI.00694-18 (PMC6026752; doi:10.1128/JVI.00694-18)
Supplement: Supplemental material [file supp_92_14_e00694-18__index.html]

Supplemental material 

# Comparative Characterization of the Sindbis Virus Proteome from Mammalian and Invertebrate Hosts Identifies nsP2 as a Component of the Virion and Sorting Nexin 5 as a Significant Host Factor for Alphavirus Replication

## Supplemental material

- Supplemental file 1 -

  Table S1 (Host proteome coverage in all backgrounds.)

  XLSX, 79K
- Supplemental file 2 -

  Table S2 (Viral proteome coverage: structural and nonstructural polyproteins.)

  XLSX, 28K
